# Therapeutic Response and Possible Biomarkers in Acute Attacks of Neuromyelitis Optica Spectrum Disorders: A Prospective Observational Study

**DOI:** 10.3389/fimmu.2021.720907

**Published:** 2021-08-04

**Authors:** Jingqi Wang, Chunping Cui, Yaxin Lu, Yanyu Chang, Yuge Wang, Rui Li, Yilong Shan, Xiaobo Sun, Youming Long, Honghao Wang, Zhanhang Wang, Michael Lee, Shane He, Zhengqi Lu, Wei Qiu, Sha Tan

**Affiliations:** ^1^Multiple Sclerosis Center, Department of Neurology, The Third Affiliated Hospital of Sun Yat-Sen University, Guangzhou, China; ^2^Mental and Neurological Diseases Research Center, The Third Affiliated Hospital of Sun Yat-Sen University, Guangzhou, China; ^3^Clinical Data Center, The Third Affiliated Hospital of Sun Yat-Sen University, Guangzhou, China; ^4^Department of Rehabilitation Medicine, The Third Affiliated Hospital of Sun Yat-Sen University, Guangzhou, China; ^5^Department of Neurology, The Second Affiliated Hospital of Guangzhou Medical University, Guangzhou, China; ^6^Department of Neurology, Nanfang Hospital of Southern Medical University, Guangzhou, China; ^7^Department of Neurology, Guangdong 999 Brain Hospital, Guangzhou, China; ^8^Department of Medicine, Harbour BioMed Therapeutics Limited, Shanghai, China

**Keywords:** neuromyelitis optica spectrum disorders, acute attack, expanded disability status scale, prognosis, biomarkers

## Abstract

**Objective:**

To explore the outcomes of NMOSD attacks and investigate serum biomarkers for prognosis and severity.

**Method:**

Patients with NMOSD attacks were prospectively and observationally enrolled from January 2019 to December 2020 at four hospitals in Guangzhou, southern China. Data were collected at attack, discharge and 1/3/6 months after acute treatment. Serum cytokine/chemokine and neurofilament light chain (NfL) levels were examined at the onset stage.

**Results:**

One hundred patients with NMOSD attacks were included. The treatment comprised intravenous methylprednisolone pulse therapy alone (IVMP, 71%), IVMP combined with apheresis (8%), IVMP combined with intravenous immunoglobulin (18%) and other therapies (3%). EDSS scores decreased significantly from a medium of 4 (interquartile range 3.0–5.5) at attack to 3.5 (3.0–4.5) at discharge, 3.5 (2.0–4.0) at the 1-month visit and 3.0 (2.0–4.0) at the 3-month visit (p<0.01 in all comparisons). The remission rate was 38.0% at discharge and 63.3% at the 1-month visit. Notably, relapse occurred in 12.2% of 74 patients by the 6-month follow-up. Higher levels of T helper cell 2 (Th2)-related cytokines, including interleukin (IL)-4, IL-10, IL-13, and IL-1 receptor antagonist, predicted remission at the 1-month visit (OR=9.33, p=0.04). Serum NfL levels correlated positively with onset EDSS scores in acute-phase NMOSD (p<0.001, R^2^ = 0.487).

**Conclusions:**

Outcomes of NMOSD attacks were generally moderate. A high level of serum Th2-related cytokines predicted remission at the 1-month visit, and serum NfL may serve as a biomarker of disease severity at attack.

**Clinical Trial Registration:**

https://clinicaltrials.gov/ct2/show/NCT04101058, identifier NCT04101058.

## Introduction

Neuromyelitis optica spectrum disorder (NMOSD), an autoimmune inflammatory disease, is commonly seen in Asian populations. The number of patients in China is currently approximately 100,000 (1, 2). Studies have demonstrated that 90% of NMOSD patients show a remission-recurrence course (3). Notably, neurological disability can occur after one attack and accumulate with each relapse in patients with NMOSD. However, the evolving course and treatment responses in patients with acute episodes of NMOSD remain unclear. Based on a small case series and experience in other immunological diseases, intravenous methylprednisolone pulse therapy (IVMP) is currently recommended as a first-line regimen for acute onset of NMOSD (4–7). However, IVMP is effective in only 60%-80% of NMOSD patients (8, 9). For patients with a poor response to IVMP treatment, plasma exchange (PE) or a large dose of intravenous immunoglobulin (IVIg) may be effective for NMOSD attacks (10, 11). In general, there is still a lack of data on the therapeutic options for NMOSD attacks and the corresponding efficacy.

Current investigations have advocated neuroinflammatory changes in NMOSD. However, there is a paucity of data on the changes in serum cytokines and chemokines in NMOSD patients (12–15). Based on the available study, we propose that cytokines related to the activation of macrophages, effectors of T and B lymphocytes and chemokines are involved in the mechanism of NMOSD. As the neurofilament light chain (NfL) shows a potential role in the pathogenesis of NMOSD (16), we measured serum levels of these cytokines/chemokines as well as NfL to assess their usefulness as biomarkers in clinical practice.

Therefore, this prospective observational cohort trial was conducted to examine treatment responses and outcomes with new-onset and relapsing NMOSD. We also explored possible serum biomarkers of disease severity and prognosis.

## Methods

### Patients Eligibility and Study Design

We conducted a prospective, multicenter observational study in the Neurology department of four hospitals in Guangzhou, China (clinical trial registration No. NCT04101058). A prospective cohort of 100 patients diagnosed with an acute attack of NMOSD was enrolled in the study from January 2019 to December 2020. The inclusion criteria for subjects enrolled in the cohort were as follows: (1) patients were diagnosed with NMOSD based on the 2015 NMOSD diagnostic criteria of the International NMO Diagnostic Team (IPND) (17); (2) an acute attack was defined as new or worsening neurological deficits lasting for at least 24 hours and occurring >30 days after the previous attack (18); (3) the symptoms were not attributable to confounding clinical factors such as fever, infection, injury, change in mood, or adverse reactions to medications; and (4) patients were male or female and ≥ 18 years old.

The Institutional Ethical Review Board of the Third Affiliated Hospital of Sun Yat-Sen University approved this study, and the Institutional Committee approved the experiments performed on patients (ID [2018] 02-362-02). Written informed consent was obtained from all participants in the study.

### Data Collection

Neurologists from four contributing centers used a predefined standardized evaluation form to assess demographic and diagnostic data, number and dates of all acute attacks from disease onset to last follow-up, attack-related clinical features, Expanded Disability Status Scale (EDSS) scores, visual acuity, and information on attack treatment and treatment outcome from the patient records. The treatment course was defined as either 6 to 11 consecutive days of therapy with high-dose intravenous steroid therapy, 5 therapeutic PEs, 5 days of IVIg or any other therapy given at least once with the intention of ameliorating an exacerbation of NMOSD. High-dose intravenous steroids corresponded to methylprednisolone at a dose 1 g in IVMP. PE was usually applied every other day. IVMP+PE/IVIg was defined as IVMP treatment with subsequent or synchronous PE or IVIg. Patients were all recommended to start IVMP treatment except for those with contraindications. Among the patients with no or partial improvement, determined by the treating physician, treatment was escalated with PE or IVIg.

Neurologic function, including visual acuity and disability assessments, were performed at attack, at the time of discharge and at the 1-, 3- and 6-month follow-up visits. Raters assessed patients using the EDSS. Treating physicians or appropriately trained investigators assessed visual acuity with the Snellen chart.

### Outcome Measures

The primary outcomes were the changes in the EDSS scores at four consecutive time points over 6 months. We also recorded the visual acuities of affected eyes as Snellen charts and transformed them to logMAR values (19).

The second outcomes were remission rate and recurrent events during the trial. A significant remission from an attack was defined as a decrease of at least 1.0 on the EDSS from a baseline score of less than 6 or a decrease of ≥ 0.5 from a baseline score of ≥ 6 (20). The remission rates at discharge and at the 1-month visit were recorded as the short-term responses to acute therapies. We also documented recurrent events as long-term prognostic indicators within 6 months after acute management.

### Aquaporin-4 and Myelin Oligodendrocyte Glycoprotein (MOG) Antibody Assay

Serum samples from patients at onset and follow-up visits were evaluated for aquaporin-4 antibody (AQP4-Ab). AQP4-Ab was assessed using a cell-based assay (CBA) based on immunofluorescence according to the manufacturer’s instructions (Euroimmu Medizische Labordiagnostika, lübeck, Germany). And serum IgG targeting myelin oligodendrocyte glycoprotein (MOG-IgG) was also detected by a CBA in our laboratory, as previously (21, 22). Immunofluorescence assay reactions were analyzed using a Zeiss Axiovert A1 microscope (German). Positive and negative human control sera were tested in each working session.

### Serum Cytokine/Chemokine and Neurofilament Light Chain Measurements

Serum was obtained from 21 NMOSD patients who had not undergone any therapy since their attack. And serum was collected as soon as possible after these patients got hospitalized and the points of sampling were a medium of 10 (IQR 8–23) days after the onset day. The serum levels of macrophage-derived cytokines (interleukin [IL]-6 and migration inhibitory factor), Th1 cell-related cytokines (tumor necrosis factor α, interferon γ, IL-1β) and Th2 cell-related cytokines (IL-4, IL-10, IL-13, IL-1 receptor antagonist [IL-1Ra]), Th17 cell-related cytokines (IL-17A, granulocyte colony stimulating factor, IL-8 and IL-9), B cell-related cytokines (IL-21 and B-cell activation factor) and several chemokines (IFN-γ-induced protein 10, monocyte chemoattractant protein-1, 2 and 4) were analyzed by MesoScale Discovery (MSD) technology. We also determined the concentrations of serum NFL (sNfL) by a sandwich immunoassay using MSD.

### Statistical Analysis

The numerical data are presented as the medium (interquartile range, IQR) and frequency and percentage (categorical data). Categorical variables were compared using Fisher’s exact test. Numerical variables were compared using the paired Wilcoxon signed rank test. Principal component analyses were used to explore cytokine/chemokine profiling to predict the severity and outcomes of NMOSD attacks. Predictive factors of response were determined by logistic regression analysis. The results are expressed as odds ratios (ORs), 95% confidence intervals (CIs), and p-values. The associations of EDSS scores and indicators of prognosis with sNfL levels were assessed by a linear regression model or logistic regression analysis, respectively. All statistical analyses were carried out using R language (V3.6.2) and SPSS Statistics for Windows, version 22.0. (SPSS Inc., Chicago, IL, USA).

## Results

### Demographic and Clinical Characteristics of the Patients

**Figure 1:**
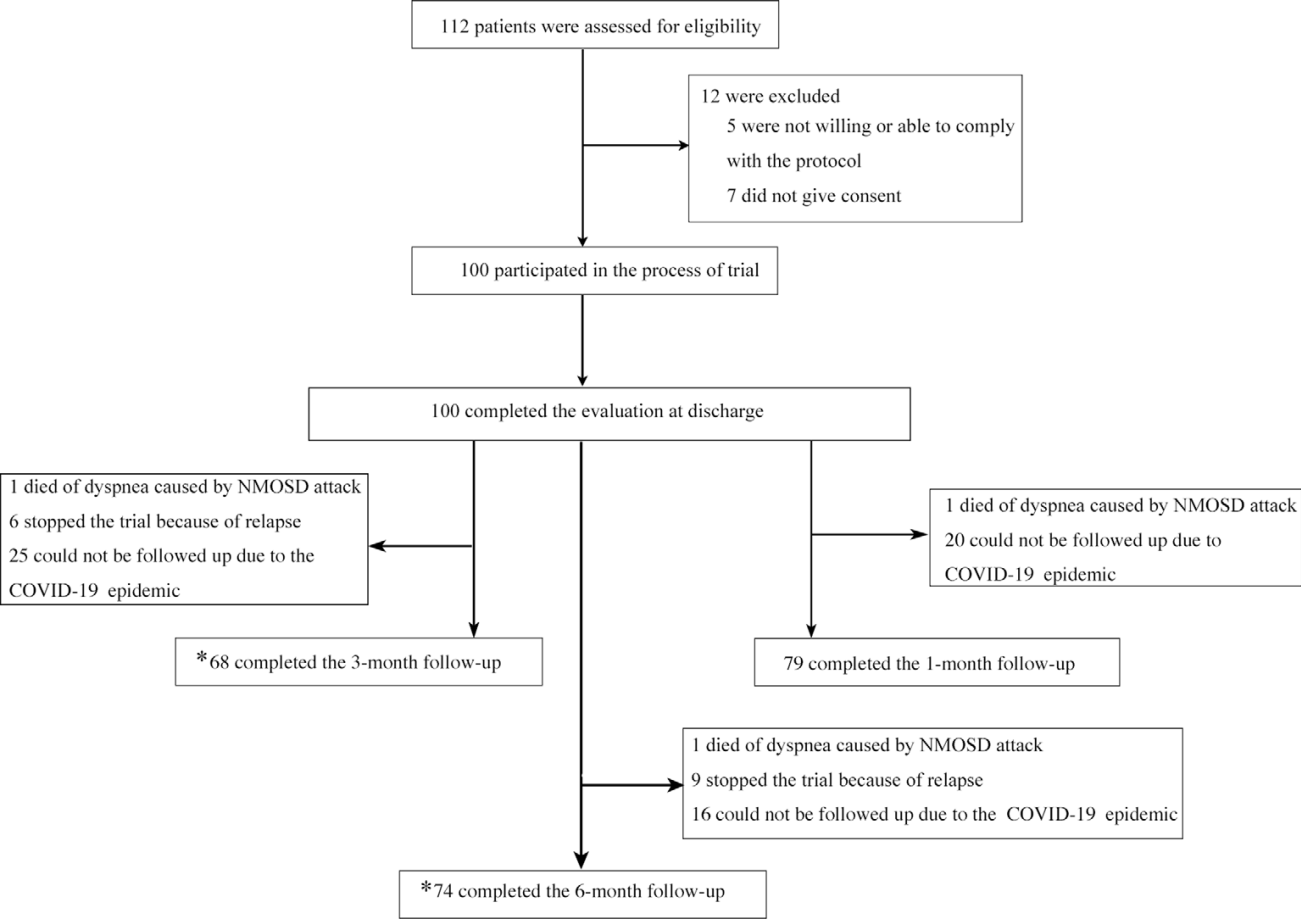
Enrollment and Follow-up. *one follow-up is counted as long as the time-point is correct regardless of whether the previous follow-up was completed or not.

**Figure 2:**
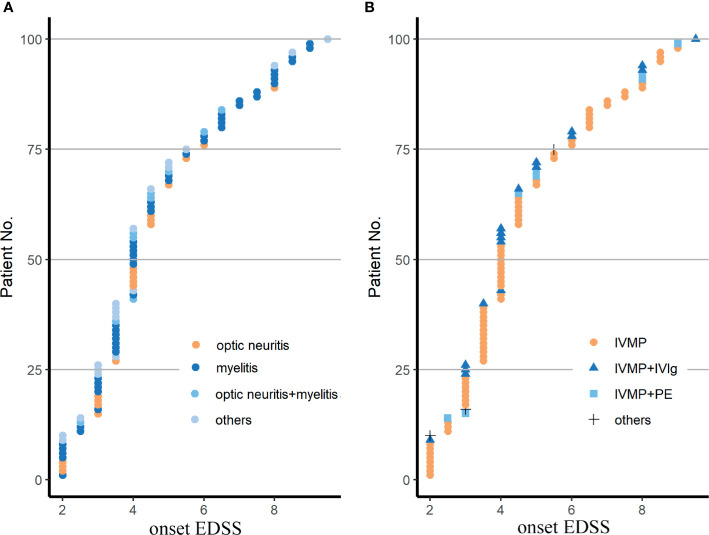
Demyelinating phenotypes **(A)** and therapeutic patterns **(B)** in the cohort. EDSS, Expanded Disability Status Scale; IVMP, intravenous methylprednisolone pulse therapy; IVMP+PE, IVMP combined with plasma exchange; IVMP+IVIg, IVMP combined with intravenous immunoglobulin; others in **(A)** included 9 patients with a brainstem syndrome, 5 with a cerebral syndrome, 6 with a diencephalic syndrome, 4 with a area postrema syndrome with or without ON or MY; others in **(B)** were 3 patients treated with oral glucocorticoid. Fisher’s exact test was used for statistical analysis.

A total of 100 patients from 4 study sites were enrolled (**Figure 1**). The baseline characteristics of these NMOSD patients are shown in **Table 1**. No difference was found in the EDSS scores among the different clinical types and therapeutic subclassifications (**Figure 2**).

**Table 1:** The baseline demographic and clinical characteristics of NMOSD patients.

	Cohort (N=100)
Gender (Female) (%)	94 (94.0)
Age at attacks, median (IQR)	43 (34–52)
Disease duration (years)^1^, median (IQR)	4.5 (2.0–7.8)
No. of relapses in previous year, median (IQR)	1 (1–2)
Annualized relapse rate (5 years), medium (IQR)	0.8 (0.4–1.2)
First attack (Yes) (%)	23 (23.0)
Manifestations of attacks (%)	
Isolated ON, NO.	20 (20.0)
Isolated MY, NO.	50 (50.0)
ON+MY, NO.	10 (10.0)
Others^2^	20 (20.0)
AQP4-IgG-seropositive status (%)^3^	81/95 (85.2%)
MOG-IgG-seropositive status (%)^3^	0/95 (0)
EDSS score, median (IQR)	4 (3–5.5)
Immunosuppressive therapy at baseline (%)	
None	55 (55.0)
Glucocorticoids alone	7 (7.0)
Azathioprine with or without glucocorticoids	17 (17.0)
Mycophenolate mofetil with or without glucocorticoids	18 (18.0)
Rituximab with or without glucocorticoids	3 (3.0)
Treatment (%)	
IVMP alone	71 (71.0)
IVMP+PE	8 (8.0)
IVMP+IVIg	18 (18.0)
Other^3^	3 (3.0)
Maintenance therapy (%)	
Dead	1 (1.0)
None	5 (5.0)
Loss to follow-up after 1 month	17 (17.0)
Glucocorticoids alone	3 (3.0)
Azathioprine with or without glucocorticoids	21 (21.0)
Mycophenolate mofetil with or without glucocorticoids	47 (47.0)
Rituximab with or without glucocorticoids	6 (6.0)

IQR, interquartile range; NMOSD, neuromyelitis optica spectrum disorder; EDSS, Expanded Disability Status Scale; AQP4-IgG, anti–aquaporin-4 antibodies; MOG-IgG, anti-myelin oligodendrocyte glycoprotein antibodies; ON, optic neuritis; MY, myelitis; ON+ MY, ON combined with MY; IVMP, intravenous methylprednisolone pulse therapy; IVIg+PE, IVMP combined with plasma exchange; IVMP+IVIg, IVMP combined with intravenous immunoglobulin.

^1^Disease duration was not available in newly onset NMOSD patients (n = 23).

^2^Included 9 with a brainstem syndrome, 5 with a cerebral syndrome, 6 with a diencephalic syndrome, 4 with a area postrema syndrome with or without ON or MY in the whole cohort; 7 with a brainstem syndrome, 5 with a cerebral syndrome, 4 with a diencephalic syndrome, 2 with area postrema syndrome with or without ON or MY in the IVMP group.

^3^Five patients did not perform any autoimmune antibody tests at attack point.

^4^Treated with oral glucocorticoid.

### The Outcome of Attacks of NMOSD Was Generally Moderate

**Figure 3:**
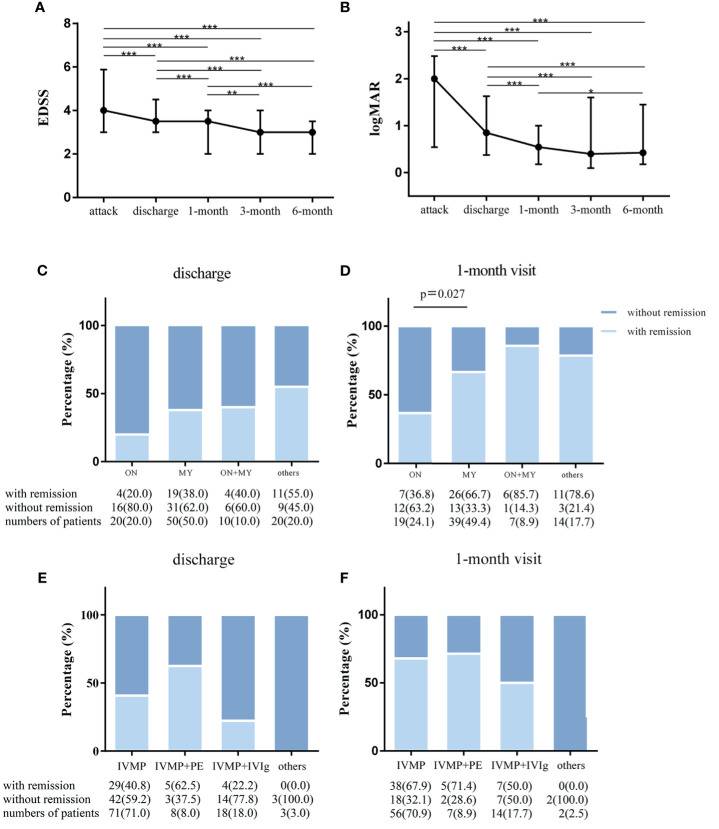
The outcome of attacks of NMOSD patients. Changes in expanded disability status scale (EDSS) scores and visual acuities in NMOSD patients from acute attacks to 6-month follow-up. **(A)** EDSS scores were compared in all matched patients corresponding to 4 time points in the whole cohort. **(B)** The values of visual acuities of the affected eyes were recorded as Snellen charts and transformed to logMAR values in patients with optic neuritis attacks. Paired Wilcoxon signed rank test was used for statistical analysis; and remission rates categorized by clinical manifestations and therapeutic methods in the cohort. **(C)** Remission status at discharge (n = 100) and **(D)** at the 1-month visit (n = 79) in isolated myelitis (MY), isolated optic neuritis (ON), simultaneous MY plus ON (MY+ON) and other subtypes. **(E)** Remission rates at discharge (n = 100, **E**) and at the 1-month visit (n = 79, **F**) with treatment with intravenous methylprednisolone pulse therapy (IVMP), IVMP combined with plasma exchange (IVMP+PE) IVMP combined with intravenous immunoglobulin (IVMP+IVIg) and other drugs. Fisher’s exact test was used for statistical analysis. *p < 0.05, **p < 0.01, ***p < 0.001.

The EDSS score at discharge and at a follow-up of 1, 3, and 6 months decreased significantly compared to that at the attack point (p<0.001 in all comparisons, **Figure 3A**). The EDSS score of each point was significantly lower than that of the previous point, except for the 6-month and 3-month comparisons (p<0.001, discharge vs 1-month visit; p<0.01,1-month vs 3-month visit; p<0.001,1-month vs 6-month visit; p=1,3-month vs 6-month visit, **Figure 3A**). Numerically, the EDSS score decreased modestly but statistically significantly from 4.0 (3.0–5.5) at attack to 3.5 (3.0–4.5) at discharge, to 3.5 (2.0–4.0) at the 1-month visit and to 3.0 (2.0–4.0) at the 3-month visit.

Regarding the logMAR visual acuity of affected eyes in optic neuritis (ON) attacks, there was remarkable visual improvement at discharge compared to the onset point (n=44 (affected eyes), 0.85[0.37–1.63] vs 2[0.54–2.48], p<0.001), followed by a small magnitude of recovery at 1 month compared to the point of discharge (0.54 [0.18–1.00] vs 0.85[0.37–1.63], n=38, p<0.001, **Figure 3B**). In addition, a statistically significant visual improvement increase was observed at 6 months (0.47[0.18–1.45] vs 0.54[0.18–1.00], n=30, p<0.05), but no significant recovery was obtained at the 3-month visit (0.40 [0.1–1.60] vs 0.85[0.37–1.63], n=34, p=0.207) when compared to the 1-month visit (**Figure 3B**).

We also assessed the clinical outcome by remission rates grouped by clinical manifestation and treatment option. The remission rate was similar across the 4 submanifestations at the discharge point (**Figure 3C**). However, remission rates were significantly lower for isolated ON than for isolated myelitis (MY) at the 1-month visit (36.8% vs 66.7%, p=0.027, **Figure 3D**). Irrespective of clinical manifestations, no significant difference was found among the four treatment options for NMOSD attacks (**Figures 3E, F**), and this finding was likely due to the small numbers of cases in some subgroups.

### Frequency and Manifestation of Relapses During the 6-Month Follow-Up

Nine patients in the group (n=74) experienced another relapse during the 6-month follow-up. The patients’ clinical and laboratory profiles are summarized in **Table 2** and revealed various transforms of phenotypes, therapeutic patterns and AQP4-Ab status in these patients.

**Table 2:** Clinical characteristics of 9 NMOSD patients who experienced recurrences during the 6 months.

	Case1	Case2	Case3	Case4	Case5	Case6	Case7	Case8	Case9
Gender	F	F	F	F	F	F	F	M	M
Date at onset	19/2/11	19/3/21	19/3/20	19/5/16	19/8/16	19/8/16	19/1/20	20/3/11	19/10/15
AQP4 titer at attack^1^	1:100	1:100	1:32	1:320	1:320	Negative	Negative	ND	ND
EDSS score before attack	0	3.5	4.5	3.5	4	2.5	2	1	0
EDSS score at attack	6	4	6.5	3.5	4.5	4.5	4	2	4
Phenotype of lesion at attack	ON+ MY	ON+ MY	MY	MY	ON	MY+ brainstem	brainstem	ON	ON+ MY
Treatment at attack	IVMP+ IVIg	IVMP	IVMP	IVMP	IVMP+ PE	IVMP	IVMP	IVMP	IVMP
Immuno-therapy after acute phase	MMF	RTX	MMF	MMF	MMF	MMF	None	MMF	MMF
Date at relapse	19/5/4	19/8/4	19/5/22	19/11/29	19/10/10	19/12/15	19/06/15	20/7/16	19/10/15
EDSS score before-relapse	3.5	3.5	6	3	4.5	3.5	3.5	2	3
AQP4-IgG titer before relapse	1:32	1:320	1:10	1:320	1:320	Negative	Negative	ND	1:32
EDSS score at relapse	4	6.5	7	3	4.5	6	4	2.5	3
Phenotype of lesion at relapse	MY	MY	MY	ON	Area postrema	MY	ON+MY	ON+MY	ON

AQP4, anti–aquaporin-4 antibodies; EDSS, Expanded Disability Status Scale; ON, optic neuritis; MY, isolated myelitis; ON+MY, ON combined with MY; IVMP, intravenous methylprednisolone pulse therapy; IVIg+PE, IVMP combined with plasma exchange; IVMP+IVIg, IVMP combined with intravenous immunoglobulin; MMF, azathioprine; RTX, rituximab; ND, no data.

^1^These 9 patients were seronegative for MOG-IgG except for case 8 who did not get tested.

### T Helper Cell 2 (Th2)-Derived Cytokines Were Predictors of Remission From NMOSD Attacks at the 1-Month Visit

**Figure 4:**
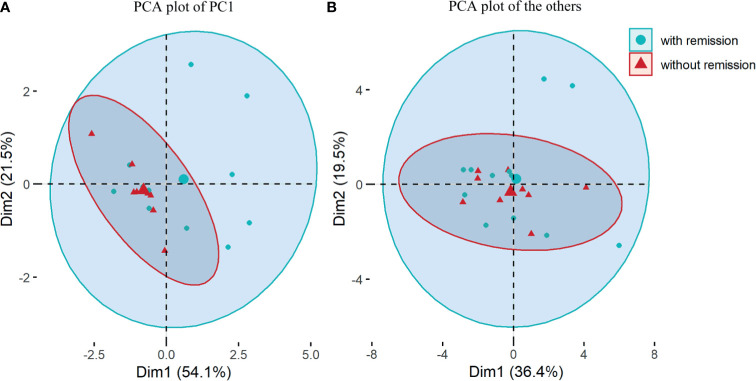
Principal component analysis (PCA) based on 19 multiple cytokines and chemokines. **(A)** PCA plot for principal component (PC)1 composed of interleukin(IL)-4, IL-10, IL-13 and IL-1 receptor atagonist; **(B)** PCA plot for 15 other cytokines and chemokines including rotated components (RC)1, RC2 and RC3. Comparisons were between patients with remission and those without remission from NMOSD attacks at 1-month visit.

The concentrations of nineteen cytokines and four chemokines are shown in **Supplemental Table 1**. We explored serum cytokine/chemokine profiling to predict disease severity and outcomes. Based on principal component analysis (PCA) results presented as scree plots (**Supplemental Figure**), 19 cytokines and chemokines were divided into 4 categories: IL-4, IL-10, IL-13 and IL-1Ra were synthesized as one principal component 1 (PC1), and the other 15 cytokines were grouped into three other rotated components (RCs) named RC1, RC2 and RC3. The onset EDSS scores (over 6 or not), remission status at discharge/1-month visit and recurrent events at the 6-month follow-up were regarded as dependent variables to complete PCA. These 4 components were fitted to a multivariate logistic regression model. The results showed that PC1 was an independent factor for remission at 1 month (OR=9.33, 95% CI 1.60–147.14, p=0.044, **Table 3** and **Figure 4**). An attack tends to be relieved at the 1-month visit when the PC1 value increases, and the contribution coefficient of PC1 to the outcome is 2.23 (**Table 3**). The coefficients of the cytokines, including IL-4, IL-10, IL-13 and IL-1Ra, ascribed to PC1 are presented in **Supplemental Table 2**.

**Table 3:** Combined cytokines associated with remission from NMOSD attacks at 1-month visit.

	*β*	*Odds Ratio* (9*5%*CI)	*p* value
PC1	2.23	9.33 (1.60–147.14)	0.044
RC1	-0.89	0.41 (0.07–1.57)	0.236
RC2	-0.94	0.39 (0.06–1.87)	0.268
RC3	-0.15	0.86 (0.19–19.28)	0.881

Logistic multivariate analysis based on four components.

20 cytokines and chemokines were divided into principal component1 (PC1), and other three principal components (RCs) named rotated components (RC1, RC2 and RC3). CI, confidence interval.

### Serum Neurofilament Light Chain Levels Were Positively Related to the Severity of Disability of an NMOSD Attack

**Figure 5:**
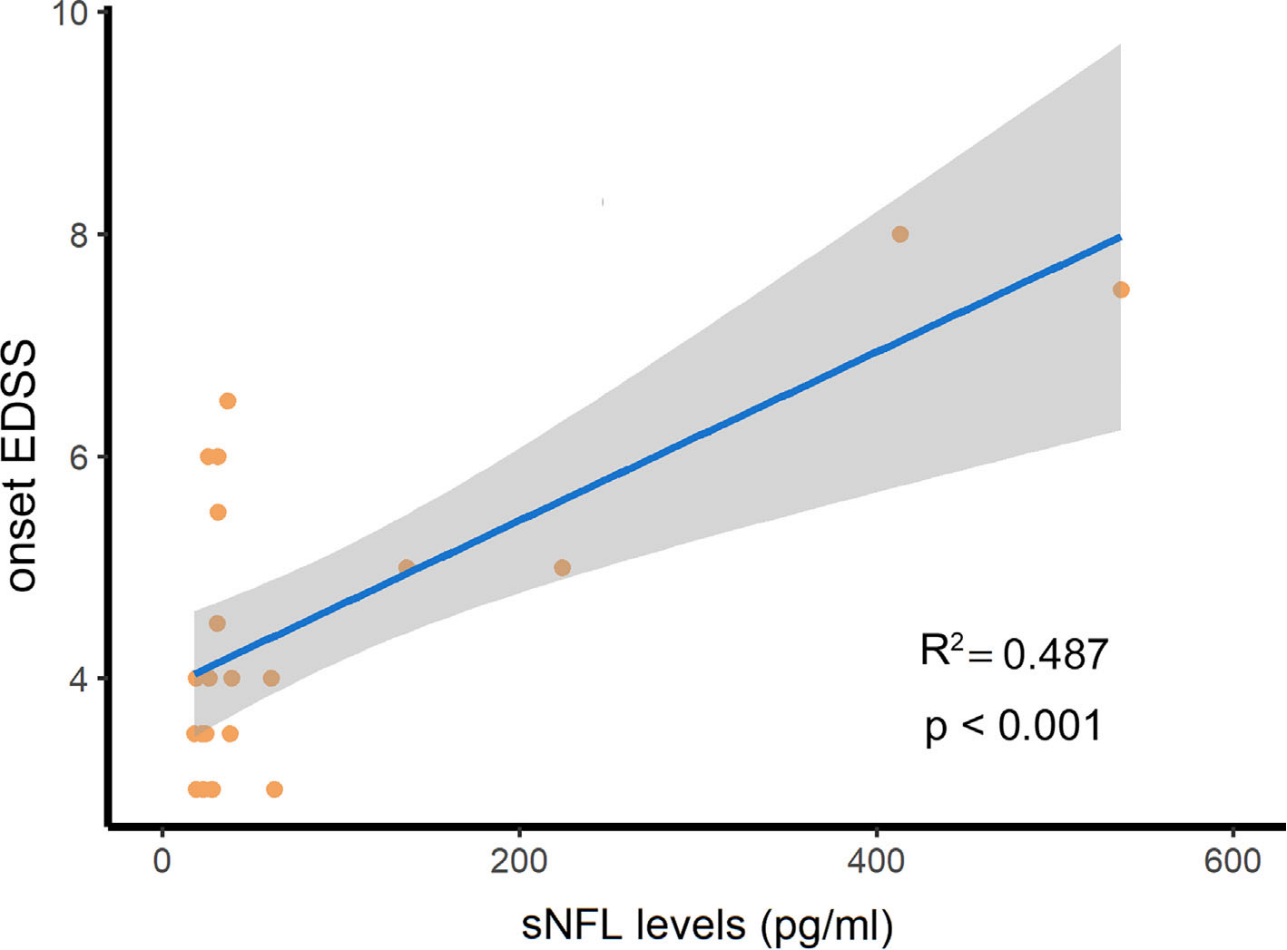
The serum NFL (sNFL) levels were positively related with the expanded disability status scale (EDSS) scores at attack in NMOSD patients. R2, determination coefficient.

The sNFL levels were 31.1 (24.4–60.9) pg/ml in 21 NMOSD patients who had not received the therapies after their attacks. We assessed the association between sNfL levels and disease severity and indicators of prognosis of NMOSD. As a result, sNfL levels in NMOSD patients in the acute phase were positively correlated with EDSS scores at attack (R^2 =^ 0.487, p<0.001, **Figure 5**). This means that NMOSD patients with more severe attacks are prone to have higher levels of sNfL at the onset stage. However, there was no difference in sNfL levels between the groups divided by therapeutic responses at discharge or at the 1-month follow-up or recurrence or not during the next 6 months of follow-up.

## Discussion

This is a prospective study that recruited NMOSD patients in the acute phase to observe the characteristics and outcomes of attacks. In terms of subtype classification, MY accounted for the most cases, followed by ON and brain/brainstem syndromes, which is in line with previous studies (8, 23). Regarding therapeutic options, a majority of patients (71%) were treated with IVMP alone, and only 8% opted for PE in this trial, exhibiting a notable difference from other studies. Apheresis therapies such as PE and immune adsorption account for 20% or more of acute treatments in other studies (8, 24). The reasons are complicated and are mainly due to the relatively mild severity of the patients in our cohort.

In the whole cohort, the EDSS scores continuously and significantly decreased until the 3-month visit (3.0 [2.0–4.0]) compared with the onset scores (4.0 [3.0–5.5]). Since EDSS is insensitive to vision change, we use logMAR to evaluate the improvement of vision defect (25). The visual improvement reached a plateau at 1 month and could not be further improved until the 6-month point, which was partially consistent with the EDSS scores. We then explored the remission rate in all patients. Thirty-nine percent (38%) of the subjects showed remission at discharge, and 63.3% showed remission at 1 month after acute remedy. Overall, the outcome of attacks was generally moderate. This is fractionally different from other cohorts with NMOSD attacks. A total of 21.6% of NMO attacks showed a full recovery, and 6% showed no improvement at all after the acute treatment course (8). Suitable outcome criteria designed for clinical symptoms in NMOSD attacks are scarce, and the formula we applied in this study based on the degree of disability (20). Hence, the variability of outcome between this and other studies may be attributed to the differences in the evaluation criteria and the condition of the enrolled population.

We observed the efficacy measured by remission rate across phenotypes. Attacks such as ON, MY, and ON combined with MY and other classifications had the same remission rate at discharge. Of note, isolated ON was associated with particularly unfavorable outcomes and attacks involving MY had a higher likelihood of achieving remission at the 1-month follow-up. This might indicate that the recovery of vision impairment is slower than that of paralysis, sensory loss and bladder dysfunction. Differently, manifestation as MY or bilateral ON was associated with particularly unfavorable outcomes after the course of therapies among NMO patients in Germany (8). We also compared the efficacy of different treatment options, but no significant difference was found. Although studies ascertained the importance of PE as a therapeutic intervention in acute attacks (10, 26), PE was not associated with an additional curative effect when compared to IVMP alone in our trial. These might be due to the limited number of cases in some treatment options and/or possible baseline differences between distinct subgroups, an inherited disadvantage of an observational study design. In fact, PE is recommended as soon as possible for patients with severe attacks of CNS demyelination, with or without a combination of IVMP (27, 28).

The present study provides data on a second clinical event following acute NMOSD attacks in a relatively short period. In detail, approximately 9% of patients relapsed within 3 months, and 13% relapsed within 6 months during this trial. And the previous annualized relapse rate of this cohort was 0.8(IQR 0.4–1.2) per year, which seemed higher than that in follow-up phase after this episode. And we might ascribe this phenomenon to the better compliance of the patients after enrollment. From a long-term perspective, approximately 60% of patients relapse within 1 year, while 90% relapse within 3 years in another study (3). Scholars also reported that 86.6% of pediatric NMOSD patients had a second clinical event after a follow-up of 2–10 years, with a median disease duration of 4 years (29). Those patients who experienced recurrence in our trial had no special attributes and were not clustered in onset clinical subtype, AQP4 status or treatment modality. A recent study suggested that approximately half of relapses occurred within 12 months and presented with similar manifestations as the last clinical attack (23).

B-cell modulation, imbalance of Th1/Th2 and upregulation of Th17 are essential factors for developing NMO inflammatory lesions (30, 31). Cytokines, including IL-4, IL-10, IL-13 and IL-Ra, are Th2-type cytokines and are beneficial in CNS inflammatory disorders (13, 32). Our study reveals that a high level of Th2-type cytokines (PC1) composed of these four cytokines rather than other components predicts remission at the 1-month visit but not at discharge. This suggests a protective role of a Th2 dominant response in the cascade of immune events involved in NMOSD pathogenesis. Moreover, the prognostic effect was not achieved at the end of acute management, which might be attributed to the delayed effect of Th2 cells in the natural course of NMOSD attack. Interestingly, there was an increased peripheral Th2 proportion (CD4^+^IL-4^+^) in the attack phase compared with the remission phase, and the Th2 proportion was associated with multiple lesion locations in AQP4-IgG-positive patients (33). These results both supported a potential role of Th2 cells in the pathogenesis and progression of NMOSD. More studies are required to further elucidate the immunological characteristics of NMOSD. Our study supports that the Th2 cytokine profile, including IL-4, IL-10, IL-13 and IL-Ra, might improve our ability to monitor the treatment response in acute NMOSD episodes.

NfL is a structural element of neurons that results from neuroaxonal damage and appears in CSF and blood in neurological disease (34–37). In our trial, sNfL levels were positively associated with EDSS scores but irrelevant to the degree of recovery from NMOSD relapses. In addition, we demonstrated that sNfL levels could not predict subsequent relapses within 6 months in patients with NMOSD. It was reported that sNfL levels increased alongside the EDSS scores and age and remained high for a longer time after relapse in patients with NMOSD, in line with our finding (16). Furthermore, our team also investigated sNfL level of Chinese NMOSD patients with single molecule array (SIMOA) method, a more sensitive assay, and the level of sNfL was lower [medium 7.97 (range 10.55–27.94) pg/mL] than that in this study (38). The deviation might attribute to the difference between these two cohorts. Taken together, sNfL could be a biomarker of disease activity and disability but might not influence the course of an NMOSD attack. It is noteworthy sNfL showed predictive value for long-term clinical outcomes in multiple sclerosis and Guillain-Barré syndrome (39, 40).

In conclusion, the outcome of attacks of NMOSD was generally moderate, and ON might have poorer remission rates than MY during 1 month. Furthermore, this study identifies Th2-related cytokines characterizing the prognosis of acute episodes of NMOSD at 1 month, and sNfL is likely to be a biomarker of disease severity at attack. It might extend the clinical knowledge of the treatment response and prognosis and severity of patients with NMOSD attacks. A limitation that should be noted is that COVID-19 raised the rate of loss to follow-up, which caused small numbers of cases in some subgroups. Meanwhile, we measured serum biomarkers by MSD other than SIMOA which previous study always applied. Although the concentration of serum biomarkers included NfL and cytokines/chemokines detected in our study was tend to be consistent with that detected by SIMOA in other studies (16, 39, 41, 42), prospective studies are warranted to confirm the current findings.

## Data Availability Statement

The original contributions presented in the study are included in the article/**Supplementary Material**. Further inquiries can be directed to the corresponding author.

## Ethics Statement

The studies involving human participants were reviewed and approved by The Institutional Ethical Review Board of the Third Affiliated Hospital of Sun Yat-Sen University, and the Institutional Committee approved the experiments performed on patients (ID [2018] 02-362-02). The patients/participants provided their written informed consent to participate in this study.

## Author Contributions

JW and CC contributed to the data acquisition, drafting of the manuscript, statistical analysis, and technical and material support. YC, YW, YML, HW, ZW and RL were involved in technical support and participant enrolment. XS contributed to measure AQP4-IgG levels and multiple cytokines and chemokines in serum. YXL and YS contributed to the analysis and interpretation of data and technical support. ML and SH was involved in participant enrolment and technical and material support. ZL and WQ contributed to design and conceptualized study, interpreted the data, revised the manuscript for intellectual content. ST was involved in the study concept and design, critical revision of the manuscript, procurement of funding, study supervision, and final approval of the version to be published. All authors contributed to the article and approved the submitted version.

## Funding

This study was funded by China Postdoctoral Science Foundation (2018M643335), the Fundamental Research Funds for the Central Universities (2021qntd34) and Guangdong Science and Technology Department-Regional Joint Fund (2020A1515111133).

## Conflict of Interest

Authors ML and SH were employed by company Harbour BioMed Therapeutics Limited.

The remaining authors declare that the research was conducted in the absence of any commercial or financial relationships that could be construed as a potential conflict of interest.

## Publisher’s Note

All claims expressed in this article are solely those of the authors and do not necessarily represent those of their affiliated organizations, or those of the publisher, the editors and the reviewers. Any product that may be evaluated in this article, or claim that may be made by its manufacturer, is not guaranteed or endorsed by the publisher.
