# Supplementary material for: Therapeutic Response and Possible Biomarkers in Acute Attacks of Neuromyelitis Optica Spectrum Disorders: A Prospective Observational Study
Source: Front Immunol. 2021 Aug 4;12:720907. doi: 10.3389/fimmu.2021.720907 (PMC8372759; doi:10.3389/fimmu.2021.720907)
Supplement: Supplementary file 1 [file DataSheet_1.doc]

***Supplementary Material***

# Supplementary Figures and Tables

## Supplementary Figures


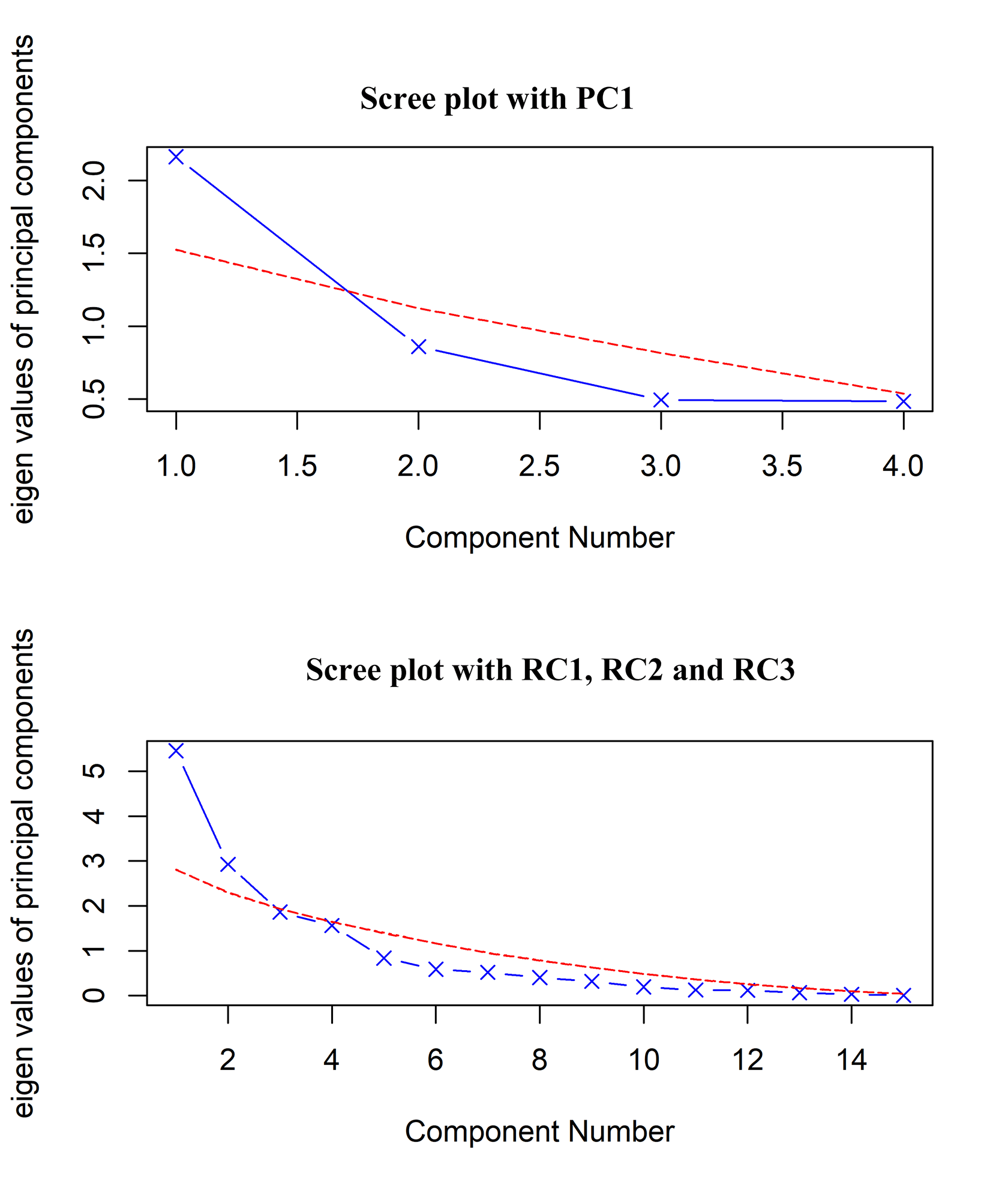


**Supplementary Figure 1.** Scree plots from principal component analysis based on 19 cytokines and chemokines. Interleukin (IL)-4, IL-10, IL-13 and IL-1 receptor atagonist were synthesized as principal component 1 (PC1) and the other 16 cytokines were divided as three other rotated components (RCs) named RC1, RC2 and RC3.

## Supplementary Tables

# Supplemental Table 1. The concentrations of fifteen cytokines and four chemokines in serum of NMOSD patients at onset stage.

|  | Concentrations (pg/ml)  medium (IQR) |
| --- | --- |
| IL-6 | 1.02 (0.74–1.65) |
| migration inhibitory factor | 65828 (45797–88409) |
| tumor necrosis factor-α | 4.22 (3.29–5.27) |
| interferon γ | 13.1 (8.26–20.4) |
| IL-1β | 0.24 (0.19–0.33) |
| IL-4 | 0.07 (0.06–0.09) |
| IL-10 | 0.44 (0.20–0.58) |
| IL-13 | 2.71 (1.73–3.60) |
| IL-1 receptor antagonist | 146 (115–309) |
| IL-17A | 1.92 (1.23–2.68) |
| granulocyte colony stimulating factor | 5.38 (4.01–6.52) |
| IL-8 | 7.93 (5.25–10.5) |
| IL-9 | 0.12 (0.08–0.17) |
| IL-21 | 0.82 (0.31–1.85) |
| B-cell activation factor | 423 (287–588) |
| IFN-γ-induced protein 10 | 141 (70.0–183) |
| MCP-1 | 82.6 (63.1–183) |
| MCP-2 | 16.1 (10.8–25.4) |
| MCP-4 | 116 (91.9–254) |

IL, Interleukin; MCP, monocyte chemoattractant protein

**Supplemental Table 2.** The contributive coefficient of interleukin (IL)-4, IL-10, IL-13 and IL-1 receptor antagonist (IL-1Ra) to principal component 1.

|  | coefficients |
| --- | --- |
| IL-4 | 0.331 |
| IL-10 | 0.375 |
| IL-13 | 0.376 |
| IL-1Ra | 0.267 |
